# Inactive immune pathways in triple negative breast cancers that showed resistance to neoadjuvant chemotherapy as inferred from kinase activity profiles

**DOI:** 10.18632/oncotarget.26026

**Published:** 2018-09-28

**Authors:** Takeshi Sawada, Riet Hilhorst, Savithri Rangarajan, Masayuki Yoshida, Yuko Tanabe, Kenji Tamura, Takayuki Kinoshita, Tatsu Shimoyama, Rinie van Beuningen, Rob Ruijtenbeek, Hitoshi Tsuda, Fumiaki Koizumi

**Affiliations:** ^1^ Shien-Lab, National Cancer Center Hospital, Tokyo, Japan; ^2^ Department of Pathology and Clinical Laboratories, National Cancer Center Hospital, Tokyo, Japan; ^3^ Department of Breast and Medical Oncology, National Cancer Center Hospital, Tokyo, Japan; ^4^ Department of Breast Surgery, National Cancer Center Hospital, Tokyo, Japan; ^5^ Division of Clinical Research Support, Tokyo Metropolitan Cancer and Infectious Diseases Center, Komagome Hospital, Tokyo, Japan; ^6^ PamGene International BV, 's-Hertogenbosch, The Netherlands; ^7^ Department of Basic Pathology, National Defense Medical College, Saitama, Japan

**Keywords:** triple negative breast cancer, neoadjuvant chemotherapy, tyrosine kinase activity, tumor infiltrating lymphocytes, peptide micro array

## Abstract

About 5% of Triple negative breast cancer patients (TNBCs) who receive neoadjuvant chemotherapy (NAC) experience progressive disease (PD). Few reports are published on TNBCs with PD during NAC, whereas TNBCs that respond to NAC have been well-studied. We investigated kinase activity profiles of TNBCs to explore the biological differences underlying the lack of response to NAC.

Among 740 TNBCs, 20 non-responders were identified. Seven non-responders and 10 TNBCs that did not receive NAC (control group) were evaluated. No correlation was observed between NAC response and age, menopausal status, tumor size and axillary lymph node status. Tyrosine kinase activity profiles of TNBC primary tissues from NAC non-responders and the controls were determined with a peptide microarray system. Kinase activity measurements showed that 35 peptides had significantly (*p* < 0.05) lower phosphorylation in non-responders. ZAP70, LCK, SYK and JAK2 were identified as differentially active upstream kinases. Pathway analysis suggested lower activity in immune-related pathways in non-responders. The number of tumor infiltrating lymphocytes (TILs) was significantly lower (*p* = 0.0053) in non-responders.

Kinases related to the immune system are less activated in non-responders. TILs evaluation suggested that the immune system is hardly active in non-responders and is not activated by NAC treatment.

## INTRODUCTION

Triple negative breast cancer (TNBC) is defined by the absence of estrogen and progesterone receptors and by a negative human epidermal growth factor receptor 2 (HER2)–status. As TNBC patients (TNBCs) have no indications for endocrine therapy or HER2 inhibitors, chemotherapy is the standard treatment. Neoadjuvant chemotherapy (NAC) is also used in early stage TNBCs to reduce the size of the primary tumor before surgery, and contributes to breast conservation. TNBC is a heterogeneous disease so various TNBC subgroups may show differential responses to treatment [1]. About 30% of TNBCs will achieve pathologic complete response (pCR) [2], whereas around 5% may have disease progression [3]. For this low percentage, tumor upstaging may make breast conservation or even operability impossible. Since achieving a pCR confers a survival advantage [2], many researchers have investigated clinical and molecular features of breast cancer patients with pCR. However, little has been reported on the biological characteristics of TNBC with progressive disease during NAC treatment because of the small number of cases. For better patient management, a better understanding of this is essential.

The present study was performed to reveal the biological characteristics of notably non-responders to NAC treatment by the analysis of kinase activity profiles of tumor tissue lysates. Recent technological development enables a rapid, comprehensive evaluation of kinase activities with only a few micrograms of fresh frozen tissue using peptide micro-arrays. The method has been successfully applied as prognostic [4], or predictive method [5, 6] and for target discovery [7–9] and therewith has been shown to reflect the consequences of complicated biological responsiveness. We focused on the activity profiles of tyrosine kinases (TKs) in tumor tissue including tumor cells and surrounding cells. Not only in immune therapy but also in conventional anticancer chemotherapy, accumulating evidence indicates that the antitumor activities of drugs must be attributed to the host immune system [10]. Thus, we considered that the evaluation for tumor tissues reflecting also the host immune system is important for studying the biological characteristics related to NAC response. Cellular responses to the environment including immune reaction are mediated through complex networks of signal transduction pathways driven by TKs. Therefore the activity profiles of TKs might to be associated with the response to NAC.

## RESULTS

### Clinical characteristics of patients and association with NAC response

After routine central assessment of ER, PgR, and HER2 of breast cancer patients in NCCH from January 1990 to March 2011, 740 patients were identified as TNBC. Among them, 20 patients showed clinically progressive disease (PD) after NAC treatment. PD was based on the imaging evaluation that is provided in the RECIST guideline [11]. Pathological inspection of surgical specimen confirmed non-response to NAC. Out of the 20 confirmed NAC non-responders, fresh frozen tissue was available for 7 patients only. In addition, fresh frozen tissue of 10 TNBC that did not receive NAC (controls) was obtained. Two of the seven non-responders were confirmed PD after completion of NAC. The other five patients were already confirmed as PD during the chemotherapy period, and underwent surgery before completion of the chemotherapy. The controls are assumed to be responders as 95% of patients respond to NAC [3]. The clinical characteristics of the 17 TNBC patients are detailed in Table 1. Fisher's exact test comparing proportions among controls and non-responders showed no statistical significance between NAC response and age, menopausal status, tumor size and axillary lymph node status. All patients received total mastectomy and axillary lymph nodes dissection. Median duration from the last day of NAC administration to surgery was 35 days (28–103 days).

**Table 1:** Patient characteristics

	All	Controls	Non Responder	p^*^
**Age at surgery**				
No data	1	1	0	1
<50	7	4	3	
≥50	9	5	4	
**Menopausal status**				
No data	1	1	0	1
premenopousal	6	3	3	
postmenopousal	10	6	4	
**Tumor Size (pT factor)**				
No data	1	1	0	1
pT1, pT2	6	5	1	
pT3, pT4	10	4	6	
**Number of positive lymph nodes**				
No data	1	1	0	0.145
None	5	3	2	
1–3	6	3	3	
≧4	5	3	2	
**Local treatment**				
Total mastectomy + Axillary lymph nodes dissection	17	10	7	
**Neoadjuvant chemotherapy**				
FEC+PTX			5	
AC+PTX			1	
AC+DTX			1	

Abbreviations: FEC: 5-fluorouracil, epirubicin, cyclophosphamide; PTX: paclitaxel AC: adriamycin and cyclophosphamide; DTX: docetaxel.

^*^Fisher's exact test comparing proportions among Controls and Non Responder.

### Tyrosine kinase activity profiles differ between NAC non-responders and controls

For 7 cases of TNBC patients who had pathologically confirmed non-response to NAC (non-responders) and 10 cases of TNBC patients who had received mastectomy without NAC (controls), freshly frozen breast tissue extracts were prepared and protein kinase activity was determined using the protein tyrosine kinase (PTK) PamChip^®^ arrays. For one patient in the control group, the protein content in the lysate was too low to determine protein kinase activity. Upon in depth inspection, this tissue was identified as adipose tissue and excluded from analysis. Except for this case, all breast tissue samples showed protein kinase activity. After a quality control step which eliminates peptides that show no increase in signal intensity in time, that is they are not phosphorylated by the kinases in the lysates, 82 of 142 PTK peptides were evaluated. The mean v_ini_ value per peptide for each sample was calculated and ^2^log transformed.

### Identification of peptides that show significant differences in phosphorylation between non-responder and control

**Figure 1:**
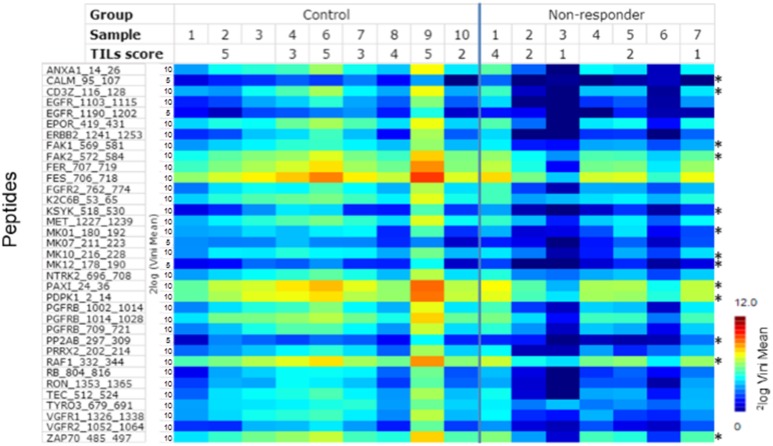
Rate of phosphorylation (Vini) for peptides that show a significant (*p* < 0.05) difference between TNBC NAC non-responders and the controls Each column represents one case, each row a peptide (for details about peptides, see Supplementary Table 1). Signal intensity is indicated by the colour scale. Peptides which derived from upstream kinases related to immune pathway are indicated by^*^.

The mean v_ini_ value for non-responders is statistically lower than for controls (*p* = 4.14 × 10^–25^, Wilcoxon's signed rank test). The controls and the non-responders were directly compared and 35 peptides were identified as statistically (*p* < 0.05, FDR = 0.090) differentially phosphorylated (Figure 1 and Supplementary Table 1).

### Putative upstream kinases

**Figure 2:**
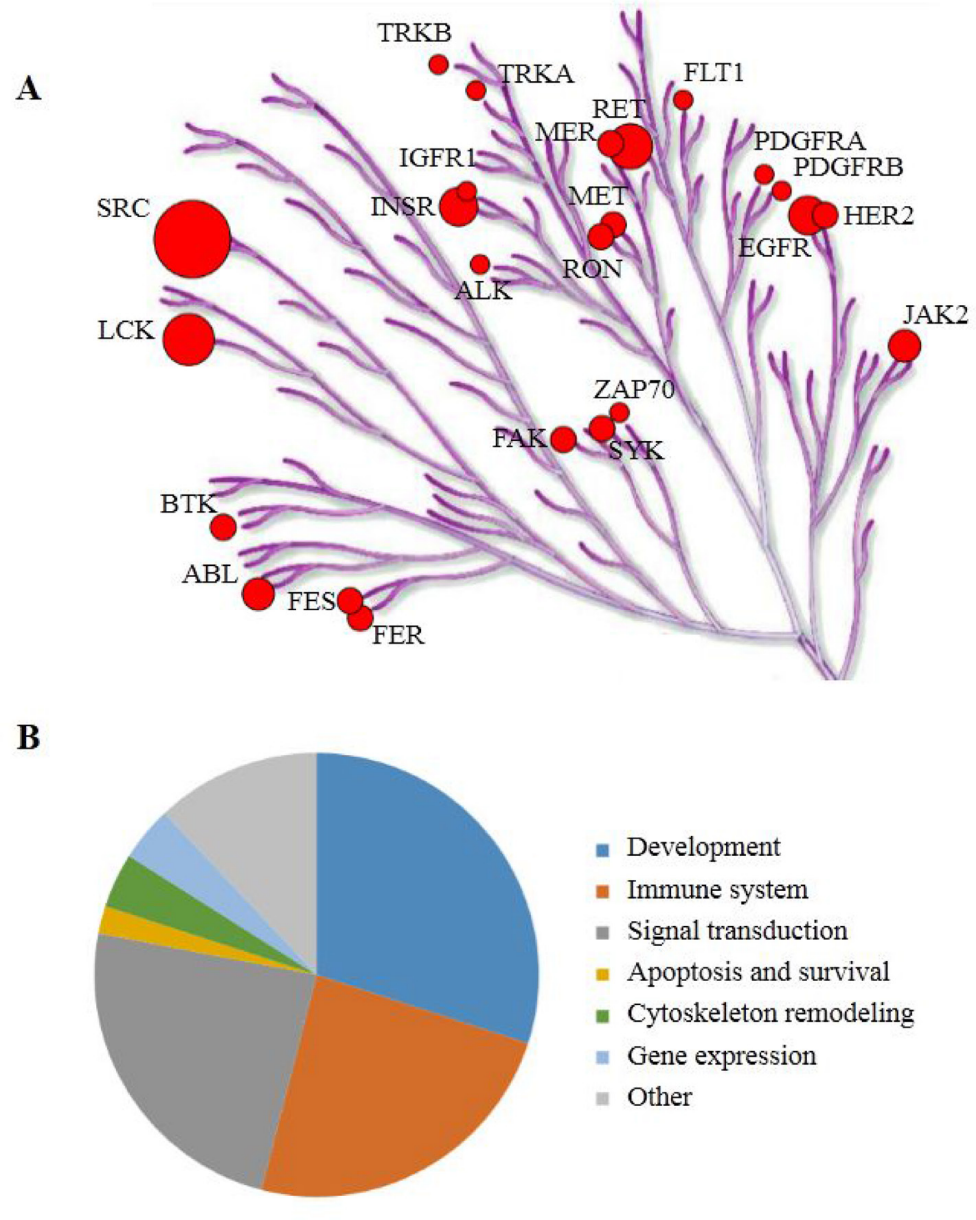
(**A**) The putative upstream kinases are projected on the phylogenetic tree of the human kinome focused on tyrosine kinases. The putative upstream kinases are generated from literature data for the 35 peptides that have statistically significant differences in phosphorylation level between non-responders and the controls. Circle size represents the number of times a kinase is mentioned as upstream kinase. Illustration reproduced courtesy of Cell Signaling Technology, Inc. (https://www.cellsignal.com/) (**B**) The 50 most significantly activated pathways grouped by processes.

For these 35 peptides, kinases reported to be able to phosphorylate the tyrosines in those peptides (upstream kinases) were identified as described in Materials and Methods. The putative upstream tyrosine kinases for each peptide are mentioned in Supplementary Table 1. Several receptor tyrosine kinases like EGFR and PDGFR were identified as well as members of the SRC family, which are downstream effectors of these receptor kinases. Furthermore, kinases involved in immune response like ZAP70, LCK, SYK and JAK2 are suggested to be differentially active. For visualization, these kinases were projected on the kinome tree (Figure 2A), using the Kinome Renderer tool.

### Pathway differences between non-responders and controls

A pathway analysis (MetaCore^TM^) using the UniProt IDs of the 35 peptides and the UniProt IDs of the proteins that contain the same sequences (as identified with Basic Local Alignment Search Tool; BLAST) yielded many pathways likely to be differently active between non-responders and controls. These pathways are highly significant, as the *p*-values for the top 50 pathways range from 3.6 × 10^–15^ to 7.3 × 10^–8^. The 50 most significant pathways were grouped according to processes (Figure 2B). Most of these pathways are involved in development and signal transduction. Also immune-related pathways show significantly lower activity in NAC non-responders. The UniProt IDs of the peptides that were projected on pathway diagrams of the three major groups are also shown in Supplementary Table 1.

### TILs in the tissue of non-responders and controls

**Figure 3:**
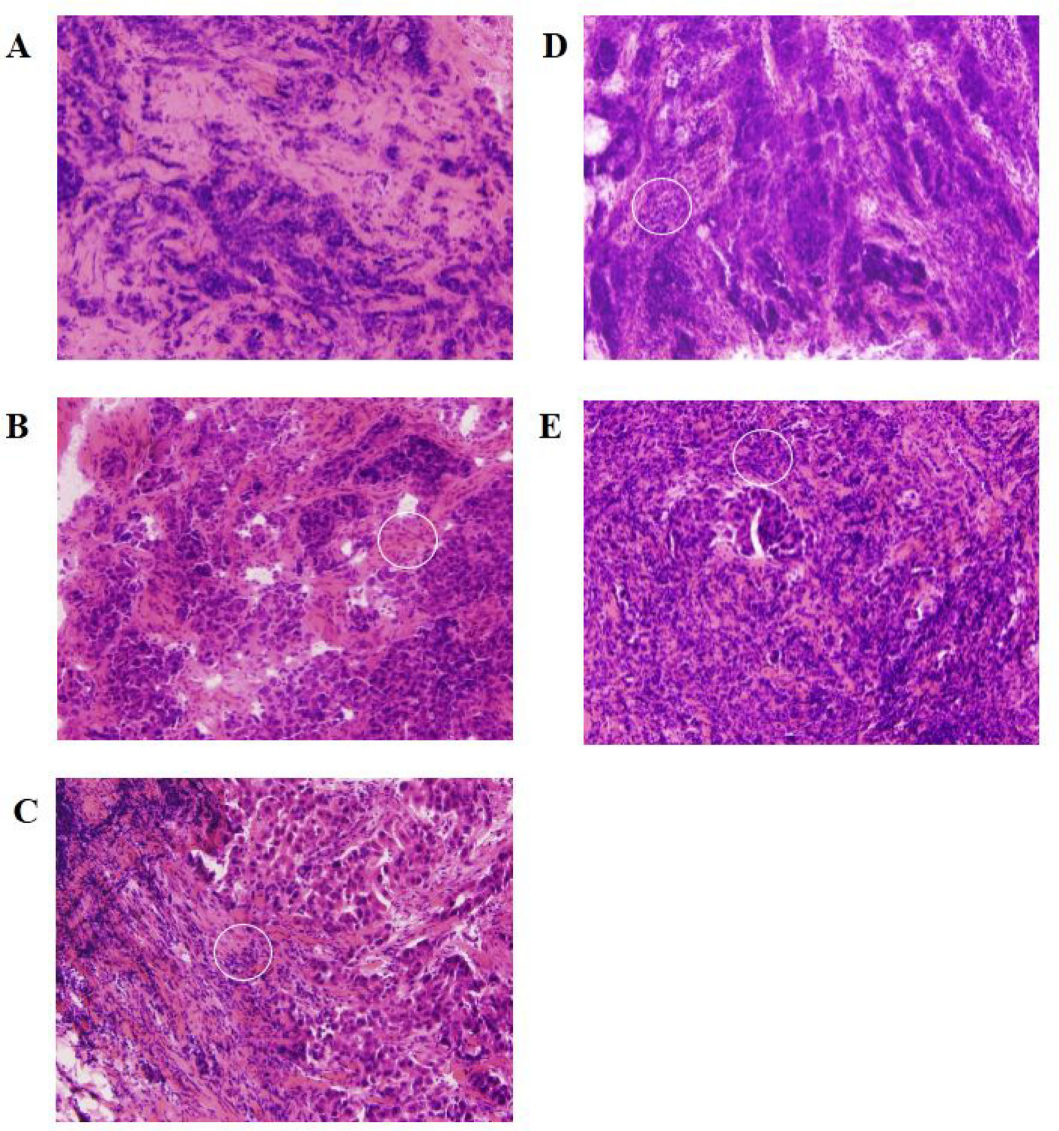
Representative photomicrographs of HE-stained tissue sections for each of the TILs scores White circle indicate TILs. TILs scores were defined as (**A**) low: 1 (≤5%), (**B**) intermediate-low: 2 (>5%, ≤10%), (**C**) intermediate: 3 (>10%, ≤40%), (**D**) high-intermediate: 4 (>40%, ≤50%), and (**E**) high: 5 (>50%).

Since both upstream kinase analysis and pathway analysis suggested a role for the immune system in response to NAC, the TILs in all samples were counted and classified on a scale of 1–5. For the non-responders, both pre and post NAC TILS scores are presented. All non-responders except one, have a low TILs score; which, for all except one, is not affected by NAC treatment (Table 2). All controls except one have a high TILs score. The median percentage of TILs was 30% (range: 5–80; Table 2). The Mann–Whitney *U* test shows that the non-responder group has a significantly lower (*p* = 0.0053) TILs score than the control group (*p* = 0.0356 using the six pre-NAC values). Figure 3 shows representative photomicrographs of HE-stained tissue sections for each of the TILs scores.

**Table 2:** TILs score of non-responders and controls

Sample		TILs Score		
		(%)		Score
Controls	1	55		5
	2	70		5
	3	60		5
	4	40		3
	5	NA		NA
	6	60		5
	7	20		3
	8	45		4
	9	80		5
	10	10		2
Non-Responder	1	45	(45)	4
	2	10	(NA)	2
	3	5	(5)	1
	4	10	(10)	2
	5	10	(5)	2
	6	10	(5)	2
	7	5	(60)	1

Figures in brackets are % of TILs in pre-NAC tissues.

### The relation between TILs score and the phosphorylation level of peptides

One-way analysis of variance (ANOVA) with TILs score as continuous factor showed that the signal on 71 peptides increased significantly with TILs score (*p* < 0.05, FDR = 0.054). The 35 peptides listed in Supplementary Table 1 are also included in this set of 71 peptides. Performing the same test with the pre-NAC TILs score (available for 6 of the 7 non-responders) yielded 67 peptides with *p* < 0.05, of which 65 were present in the set of 71 peptides. The mean signal intensity is statistically higher with increasing TILs score (*p* = 0.000763, Wilcoxon's signed rank test). Pathway analysis with these 71 peptides as input revealed an increased contribution of development and immune related pathways in the controls.

## DISCUSSION

The clinical characteristics of NAC non-responders in breast cancer are rarely investigated. Studies of clinical and biological features of non-responders can contribute to a better understanding and early identification of this patient population. Since the number of NAC non-responders in TNBC is rather small, few studies have focused on this population. Caudle *et al.* performed a study [3] with 1928 breast cancer patients who had received NAC. Fifty nine patients had PD during NAC at some point in the study. Clinical factors like tumor size, African-American race and negative hormonal receptor status were identified as independent predictors of progression in a multivariate analysis on all subtypes of breast cancer. Of the 1928 patients, 224 were TNBC, of which only 7 had PD. No sound conclusion about the TNBC population with PD could be drawn. Since factors predictive of PD are also associated with response to NAC, this study revealed the need for molecular predictors for identification of non-responders.

In the present study, we studied the biological features of TNBCs who experienced disease progression during NAC by analyzing kinase activity and linked this to infiltration of T cells. Among 740 TNBCs, we found 20 pathologically confirmed non-responders, for 7 of which fresh frozen specimens were available for further investigations. For the small number of patients in our study, clinical factors like age, menopausal status, tumor size and axillary lymph node status had no relation to response or absence of response. In this small group of samples, we found a significant difference in kinase activity between NAC non-responders and the controls that are most likely NAC-responders. In the NAC non-responders, peptides derived from receptor tyrosine kinases showed decreased phosphorylation as well as peptides derived from MAP kinases (MAPKs). A database inventory of kinases known to phosphorylate those tyrosines revealed immune related kinases and members of the SRC and MAP2K families as well as receptor tyrosine kinases. SRC and LCK are mentioned most frequently among the upstream kinases, followed by EGFR, HER2 and RET. This can suggest an important role for these kinases, but on the other hand it may reflect that kinases like SRC and EGFR are better represented in literature than kinases like e.g. BTK, FES or FER. We investigated which of these kinases are expressed in breast cell lines or tumor tissues by a focused analysis of the data published by Kothari *et al.* [12]. The relative expression of the putative upstream kinases in breast cancer cell lines and breast tumor tissue is shown in Supplementary Figure 1. Unfortunately no tumor subtypes are provided for the tissues. This figure shows that kinases like EGFR, HER2 and MET are highly expressed in cell lines, but not in tissues, making them less likely to be differentially active between non-responders and controls. Incubations with the EGFR inhibitor gefitinib showed inhibition of kinase activity in two control samples only (Supplementary Figure 2) BTK, FES, LCK, TRKA, TRKB, PDGFRB, FLT1 (VEGFR1), RET and ZAP70 kinases on the other hand are relatively high expressed in tissue as compared to cell lines. The differences in the presence of kinases between cell lines and tissues shown in Supplementary Figure 1 are most likely due to the presence of immune cells in the tissues.

Since several putative upstream kinases are immune related and also pathway analysis showed increased activity of immune related pathways in controls, TILs were counted and a high TILs score was found to be associated with a high kinase activity.

Recently, tumor infiltrating lymphocytes (TILs) have received a lot of interest as predictive biomarker for patients treated with PD-1/PD-L1 therapies in conjunction with PD-L1 expression [13]. The presence of TILs in breast cancer specimens including TNBC is now widely accepted as an important predictive and prognostic factor [14, 15]. For NAC treatment, a higher number of TILs in tumor tissue is thought to be associated with a better treatment outcome in TNBC [16]. Some retrospective studies have demonstrated that high numbers of TILs in the tumor are predictive of pCR to NAC, or associated with survival benefit in TNBCs [17]. In addition, a retrospective analysis showed that the number of TILs in residual lesions after NAC can predict patient outcome in TNBCs [18]. They found that NAC increases the number of TILs and that a higher TILs count after NAC has a better outcome. Generally chemotherapy induces immunogenic cell death which leads to the recruitment of immune-related cells including TILs by releasing high-mobility group box 1 protein (HMGB1) that promotes the cross-presentation of tumor derived antigens to T cells [19]. Therefore, TILs is an important factor not only in immunotherapy, but also in chemotherapy. Here, a significant difference in the number of TILs is observed between non-responders and controls although the statistical significance is not maintained in case one of the “controls” is not a non-responder. The controls selected for this study have not been treated with NAC, so before any treatment started they already had a high TILs count. However, a small percentage of patients with low TILs score do not respond to NAC. Data in Table 2 indicate that NAC treatment of this group does not lead to increased numbers of TILs and for one patient even caused a decrease in TILs score. A low TILs score was also reported by Tanabe *et al.* for the majority of NAC non-responders [20]. This indicates that the 7 samples analyzed here are representative for a larger population. Based on the facts, we hypothesized that immunological features differ in NAC non-responders and controls.

The present study was performed to reveal the biological characteristics of notably non-responders to NAC treatment by the analysis of kinase activity profiles of tumor tissue lysates. Furthermore we investigated whether TILs score is consistent with the difference in immune activation inferred from the kinase profiles. Kinase activity profiling suggests that receptor tyrosine kinase activity and its downstream signaling routes are activated in samples with high TILs score. More research is needed to establish whether this is due to the contribution of the TILs to the total kinase activity or to properties of the tumor cells that attract TILs or to changes in tumor cells induced by TILs. Kinase activity profiling showed that pathways related to the immune system are less activated in TNBC NAC non-responders than in controls. The lower activity of immune related kinases in non-responders implies that they have less benefit from the host immune attack on cancer cells and that this is not improved after NAC. The lower TILs score in non-responders also indicates that the immune system is less active in the elimination of cancer cells. In a gene expression-based meta-analysis, Stover *et al.* also suggested that differences in gene signatures including activation of signaling pathways and immune system related pathways may predict chemo-sensitivity in TNBC [21]. Current data show that neither TILs score nor kinase activity alone can predict the non-response to NAC. However, if kinase activity measurements could be made more specific for probing the immune related kinases by measuring the effect of addition of inhibitors of immune related kinases, it might be an alternative for counting of TILs.

This study has several limitations. The major limitation of this study is that this is a retrospective study with a limited number of patients. Second, the control group in this study is assumed to be “responders”. Among the controls, one sample had a low TILs score. Since controls were assumed to be responders based on the fact that 95% of TNBC cases is NAC responder [3], we cannot exclude that this one case would actually be a non-responder if she had been treated with NAC. So further evaluation using pretreatment samples from confirmed responders is needed to validate our findings.

Evaluation of the total number of TILs is recognized as an important prognostic biomarker. However, TILs comprise various subsets that have opposing functions against cancer cells. Recent studies revealed that the ratio between the subsets of TILs might prove to be more useful in predicting treatment response than the total TILs score [17–19]. Since it is difficult to determine the subclasses of TILs, we envisage that kinase activity profiling could provide a fast and reliable method to circumvent the determination of TILs subclasses. We found that a high kinase activity is associated with a high TILs score, but some exceptions were observed. For one non-responder (sample 1), we found a high-intermediate TILs score. Although we could not perform further evaluation regarding TILs subsets in the present study, the ratio of subsets of TILs in this case might be different from other cases. Among the controls, one was present with a low TILs score. Since controls were assumed to be responders based on the fact that 95% of TNBC cases is NAC responder, we cannot exclude that this one case would actually be a non-responder if she had been treated with NAC.

For the determination of TILs and kinase activity, tumor tissue is required, but not easily available in sufficient quantities. Recent studies have shown the possibility to predict response to targeted therapy and immunotherapy in PBMCs by determination of kinase activity [22]. In analogy with the study showing that inhibition of PBMC kinases by sunitinib correlates to response to sunitinib in RCC [5], determination of inhibition of immune related kinases in PBMC might be able to identify the NAC non-responders.

In TNBC, new therapeutic options are needed due to the lack of effective targeted therapies, especially for the NAC non-responders, but also for the NAC responders. Recently, PD-1 inhibitors have been investigated for the treatment of highly immunogenic cancers including TNBC [23]. TILs and PD-L1 expression level are known to be the predictive biomarker for response to PD-1 inhibitors. Our results shows that TILs are low in non-responders and remain low after treatment with NAC which suggests that PD-1 inhibitors might not be an effective treatment option. Recent studies indicate that PD-L1 is mainly regulated by the type II interferon receptor signaling pathway through JAK1/2 [24]. Although PD-L1 expression was not evaluated in this study, our results of differences in JAK activation level between the control and non-responder would suggests high PD-L1 expression in responders and low PD-L1 expression in non-responders. Thus, a novel treatment strategy is needed for the NAC non-responder.

In conclusion, our analyses of kinase profiles in TNBCs have demonstrated that kinases associated with pathways regulating development, signaling and the immune system are more inactive in NAC non-responders than in the controls. Evaluation of TILs has revealed lower TILs counts in non-responders compared to the controls already before NAC and the TILs score is not boosted by chemotherapy. These data suggest that the activity of the immune system is an important determinant for response to chemotherapy in TNBC. Therefore evaluation of the immune system is important for the assessment of chemotherapy response in TNBC. Further studies are required to establish whether kinase activity profiling is able to provide more detailed information about TIL subtypes and whether it may provide a method to identify non-responders to NAC.

## MATERIALS AND METHODS

### Study participants

This study was approved by the institutional review committee of the National Cancer Center Hospital (NCCH, Tokyo). This work was carried out in accordance with the Code of Ethics of the World Medical Association (Declaration of Helsinki). Fresh frozen breast tissue was obtained from the tissue bank in the pathology division of NCCH. For the NAC responders (control group), we used surgical specimens of TNBC patients who had not received NAC because around 95% of those patients are expected to be responder [3]. The tissues of TNBC NAC non-responders were surgical specimens that were pathologically confirmed as not responding to NAC. The clinical and pathological information was collected from clinical charts and pathology reports.

### Preparation of breast tissue lysates

All samples were obtained in the NCCH. 60 μm-thick fresh frozen breast tissue slices were cut from the surgical specimens. These slices were confirmed to contain tumor cells by inspecting adjacent hematoxylin-eosin stained slices. Slices were lysed and aliquoted as described previously [25]. Since kinases in lysates are susceptible to freeze-thawing [26], a new aliquot was used for every kinase activity determination.

### Protein kinase activity profiling

Kinase activity profiles were determined using the PamChip^®^ 4 protein tyrosine (PTK) peptide microarray system from PamGene International B.V. (‘s-Hertogenbosch, The Netherlands) as described previously [25]. The PamChip^®^ 4 contains 4 identical arrays. Each array is pre-printed with 142 covalently attached peptides derived from known human tyrosine phosphorylation sites. Lysates are pumped up and down through the porous array in the presence of ATP, resulting in phosphorylation of peptides by protein kinases in the lysates. The optimal sample input was determined by testing a concentration range for two out of the 17 samples. For each PTK assay, 5 μg of protein/array was used, with 400 μM ATP. For studies with the EGFR inhibitor gefitinib a final concentration was 10 μM was used. Peptide phosphorylation was monitored during the incubation by taking images every 5 minutes, allowing real time recording of the reaction kinetics.

### Signal quantification

The fluorescence signal intensity for each peptide of each image and each time point was analyzed using BioNavigator 6.2 software (PamGene International BV), a statistical analysis and visualization software tool with an App-based infrastructure (https://www.pamgene.com/en/bionavigator.htm). For each spot the local background was subtracted from the signal intensity. To determine the initial reaction rate (v_ini_), the signal minus background from the time series of each spot was fitted to an equation for exponential association y = y_0_ + S(1–e^-k(x–x0^), where y stands for the signal intensity at cycle of measurement, k is the reaction rate constant; and (x–x0) refers to the cycle number when the image was recorded. The initial velocity of peptide phosphorylation (v_ini_) is calculated from the first derivative of this equation. Visual quality control was performed to exclude defective arrays from the analysis. For the data analysis only peptides that showed an increase in signal intensity in time on at least 30% of the arrays were included in the analysis, resulting in 82 peptides.

### Statistical analysis

After averaging signal intensities of technical replicates for each sample (n ranged from 2 to 6), peptides that were significantly (*p* < 0.05) different between non-responders and controls were identified using Student's *T*-test. The false discovery rate (FDR) was calculated as described by Benjamini and Hochberg [27].

### Upstream kinase analysis

Since a peptide sequence may occur in more than one protein, the sequences of the 35 peptides with *p* < 0.05 between non-responders and the controls were analyzed with the BLAST against the Universal Protein Resource (UniProt, https://www.uniprot.org/). For the peptides present in more than one protein (sequence identity > 0.8), the relevant phosphosites in these additional proteins were included in the analysis. Kinases known to be able to phosphorylate these sites were identified in Phosphosite (https://www.phosphosite.org/), Reactome (https://reactome.org/) and Human Protein Reference database (http://www.hprd.org/) as described in [25]. Kinases were projected on the phylogenetic tree using the Kinome Render tool (http://www.biophys.umontreal.ca/nrg/NRG/KinomeRender.html).

### Pathway analysis

The sequences of the 35 peptides significantly different (*p* < 0.05) between kinase activity profiles of non-responders and the controls were blasted against the UniProt database (https://www.uniprot.org/). The UniProt IDs for proteins that had a 100% homology with the sequences were imported into MetaCore™ (Clarivate Analytics) for pathway analysis. The UniProt ID's are projected on the predefined pathways in the program and the likelihood of this occurring by chance is calculated, where a –log (*p*-value) >4 is considered significant.

### Assessment of TILs

Consecutive sections of breast tissues were used for the assessment of TILs and for protein kinase activity profiling. All HE stained slices were independently reviewed by two investigators. The whole slide was screened using a low-power field, and a region with many lymphocytes was identified. The region for evaluation was restricted to within the tumor borders [28]. We defined TILs score as the proportion of the area infiltrated by lymphocytes within the tumor itself plus the adjacent stroma, and classified the scores as low: 1 (≤5%), intermediate-low: 2 (>5%, ≤10%), intermediate: 3 (>10%, ≤40%), high-intermediate: 4 (>40%, ≤50%), and high: 5 (>50%).

## SUPPLEMENTARY MATERIALS FIGURES AND TABLES





## Abbreviations

TNBC: triple negative breast cancer
TNBCs: triple negative breast cancer patients
NAC: neoadjuvant chemotherapy
PD: progressive disease
TILs: tumor infiltrating lymphocytes
HER2: human epidermal growth factor receptor 2
pCR: pathological complete response
CTL: cytotoxic T cell
Treg: regulatory T cell
TKs: tyrosine kinases
PTK: protein tyrosine kinase
NCCH: national cancer center hospital
FEC: 5-fluorouracil, epirubicin, cyclophosphamide
AC: adriamycin and cyclophosphamide
