# Supplementary material for: Inactive immune pathways in triple negative breast cancers that showed resistance to neoadjuvant chemotherapy as inferred from kinase activity profiles
Source: Oncotarget. 2018 Sep 28;9(76):34229–39. doi: 10.18632/oncotarget.26026 (PMC6188135; doi:10.18632/oncotarget.26026)
Supplement: Supplementary file 1 [file oncotarget-09-34229-s001.pdf]

Inactive immune pathways in triple negative breast cancers that showed resistance to neoadjuvant chemotherapy as inferred from kinase activity profiles

SUPPLEMENTARY MATERIALS

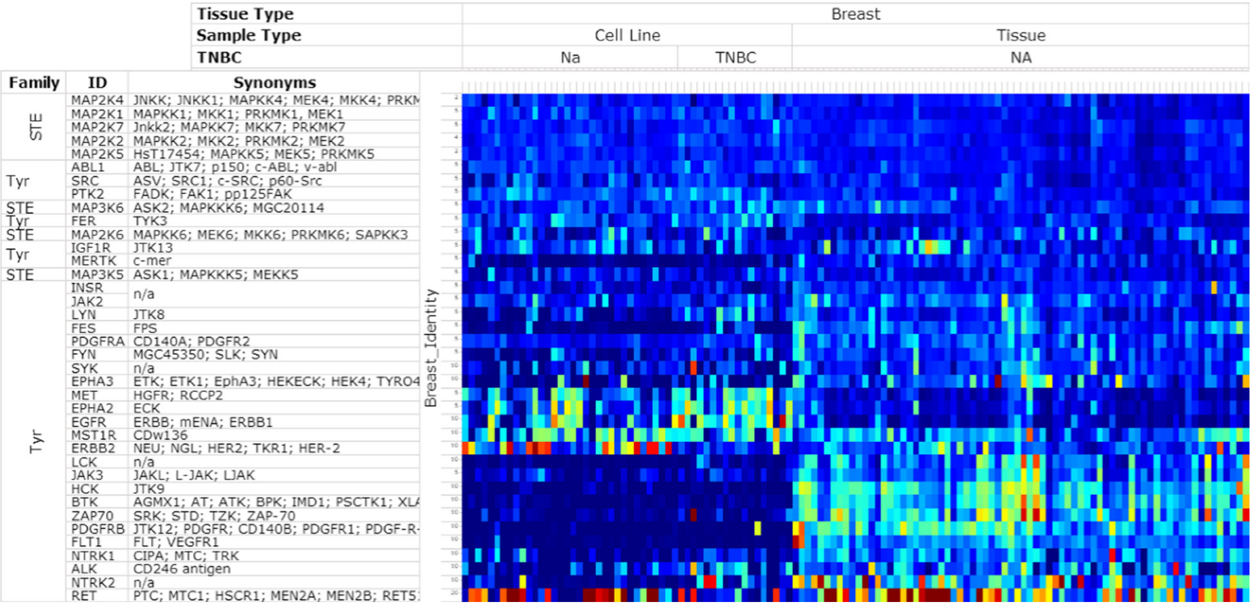

Supplementary Figure 1: Kinase expression in breast cancer cell lines and tissue. Data from Supplementary Table in Kothari *et al.* (Outlier Kinase Expression by RNA Sequencing as Targets for Precision Therapy. Cancer Discovery. 2013. https://doi.org/10.1158/2159-8290.CD-12-0336) were z-score scaled and log transformed before visualization.

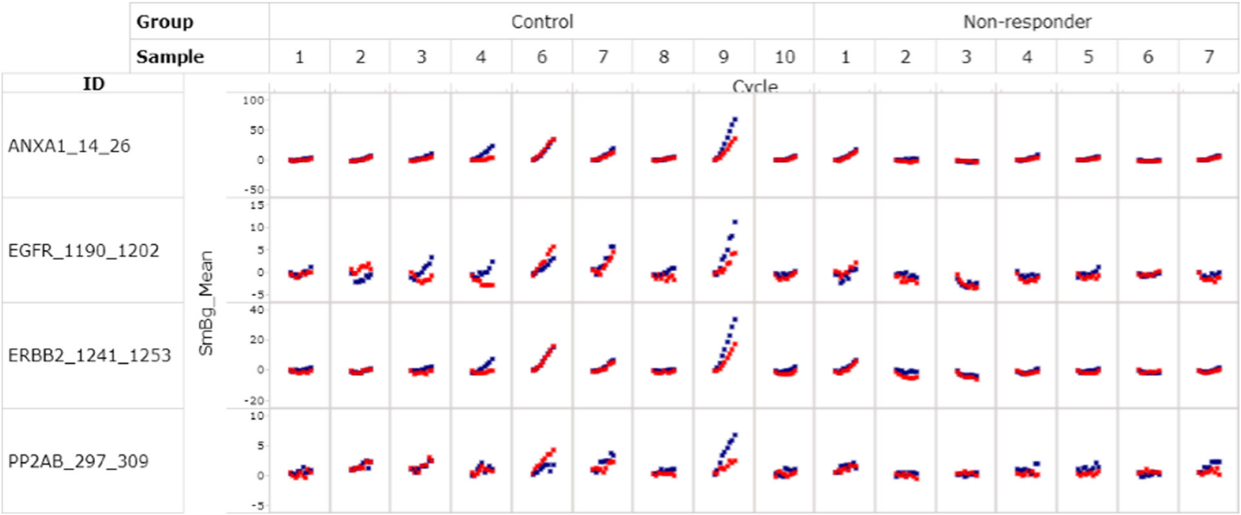

Supplementary Figure 2: Effect of EGFR inhibition. Lysates were incubated with 0.5% DMSO (blue) or 10 µM gefitinib and 0.5% DMSO (red). The peptides shown are substrates for EGFR (Table with supporting information for Figure 2).

Supplementary Table 1: The 35 peptides that were identified as statistically differentially phosphorylated between non-responders and controls, the frequency of their occurence in immune related pathways and putative upstream kinases derived from Reactome, Phosphosite and Human Protein Reference Database. See Supplementary_Table_1
